# Supplementary material for: Identifying critically ill children at high risk of acute kidney injury and renal replacement therapy
Source: PLoS One. 2020 Oct 29;15(10):e0240360. doi: 10.1371/journal.pone.0240360 (PMC7595286; doi:10.1371/journal.pone.0240360)
Supplement: S1 Table — (DOCX) [file pone.0240360.s001.docx]

**S1 Table. Day 1 Median biomarker concentrations categorized by AKI status in post-cardiac surgery subgroup**

| Day 1 values | No AKI (287) | AKI (63) | P value |
| --- | --- | --- | --- |
|  | Median (Interquartile range) | |  |
| uNGAL (ng/mL) | 36.7 (14.20, 157.00) | 81.90 (61.80, 447.00) | P = 0.12 |
| pNGAL (ng/mL) | 124.75 (115.21, 176.55) | 134. 67 (93.66, 232.95) | P = 0.38 |
