# Supplementary material for: Identifying critically ill children at high risk of acute kidney injury and renal replacement therapy
Source: PLoS One. 2020 Oct 29;15(10):e0240360. doi: 10.1371/journal.pone.0240360 (PMC7595286; doi:10.1371/journal.pone.0240360)
Supplement: S2 Table — (DOCX) [file pone.0240360.s002.docx]

**S2 Table. AUC values for biomarkers of severe AKI in post-cardiac surgery subgroup**

| **Biomarker** | **AUC (95% CI)** | **Cut Point** | **Sensitivity** | **Specificity** | **PPV** | **NPV** |
| --- | --- | --- | --- | --- | --- | --- |
| Day 1 uNGAL | 0∙53 (0∙41 0∙65) | 82.25 | 0∙67 | 0∙64 | 0.27 | 0.90 |
| Day 2 uNGAL | 0∙65 (0∙53, 0∙77) | 23.00 | 0∙68 | 0.70 | 0.48 | 0.84 |
| Day 3 uNGAL | 0∙53 (0∙37, 0∙69) | 23.00 | 0.65 | 0.60 | 0.58 | 0.68 |
| Day 1 pNGAL | 0∙70 (0∙63, 0∙77) | 139.50 | 0∙54 | 0.54 | 0.27 | 0.79 |
| Day 2 pNGAL | 0∙77 (0∙69, 0∙85) | 125. 00 | 0∙67 | 0.59 | 0.42 | 0.80 |
| Day 3 pNGAL | 0∙71 (0∙61, 0∙81) | 112. 40 | 0∙52 | 0.51 | 0.37 | 0.67 |
| RAI alone | 0∙90 (0∙86, 0∙94) | - | 0∙94 | 0.45 | 0.24 | 0.98 |
| RAI  and day 1 uNGAL | 0.81 (0.73, 0.89) | - | 0.76 | 0.71 | 0.33 | 0.94 |
| 2RAI  and day 2 uNGAL | 0.83 (0.79, 0.87) | - | 0.61 | 0.88 | 0.73 | 0.98 |
| RAI  and day 1 pNGAL | 0.58 (0.42, 0.74) | - | 0.38 | 0.76 | 0.33 | 0.79 |
| RAI  and day 2 pNGAL | 0.68 (0.52, 0.84) | - | 0.47 | 0.71 | 0.41 | 0.75 |
| RAI  and day 1 uNGAL  and day 1 pNGAL | 0.84 (0.74, 0.94) | - | 0.27 | 0.89 | 0.44 | 0.79 |
| RAI  and day 2 uNGAL  and day 2 pNGAL | 0.99 (0.95, 1.00) | - | 0.55 | 0.90 | 0.75 | 0.79 |
| RAI  or day 1 uNGAL | 0.96 (0.94, 0.98) | - | 0.96 | 0.76 | 0.40 | 0.99 |
| RAI  or day 2 uNGAL | 0.99 (0.97, 1.00) | - | 1.00 | 0.87 | 0.69 | 1.00 |
| RAI  or day 1 pNGAL | 0.97 (0.93, 1.00) | - | 0.94 | 0.76 | 0.56 | 0.97 |
| RAI  or day 2 pNGAL | 0.99 (097, 1.00) | - | 1.00 | 0.71 | 0.60 | 1.00 |
| RAI  or day 1 uNGAL  or day 1 pNGAL | 0.97 (0.87, 1.00) | - | 0.93 | 0.89 | 0.74 | 0.98 |
| RAI  or day 2 uNGAL or day 2 pNGAL | 0.99 (0.95, 1.00) | - | 1.00 | 0.90 | 0.85 | 1.00 |
