# Supplementary material for: Identifying critically ill children at high risk of acute kidney injury and renal replacement therapy
Source: PLoS One. 2020 Oct 29;15(10):e0240360. doi: 10.1371/journal.pone.0240360 (PMC7595286; doi:10.1371/journal.pone.0240360)
Supplement: S1 Appendix — (DOCX) [file pone.0240360.s003.docx]

**S1 Appendix. Development of a model for prediction of Renal Replacement Therapy**

Logistic regression and Cox proportional-hazards models were fitted to the outcome renal replacement therapy, using the independent variables where an association was found in the univariate analysis.

Independent variables associated with RRT were identified using univariate analysis. They comprised age, type of surgery, bypass time, inotrope score in first 12 hours, PELOD on day 1, lactate, base excess, platelet count, pNGAL, and procalcitonin (S3 Table).

**S3 Table. Independent variables in those children who did and did not undergo RRT.**

|  | **No RRT** | **RRT** |
| --- | --- | --- |
|  | Mean +/- Standard error of the mean | |
| Age | 3∙61 +/-0∙19 | 1∙48+/-0∙46 |
| Surgery: cardiac | 332 | 36 |
| Surgery: none | 179 | 5 |
| Surgery: other | 99 | 6 |
| Bypass time | 106∙25 +/-3∙59 | 167∙36 +/-12∙12 |
| Cross-clamp time | 63∙68 +/-2∙74 | 115∙31 +/-10∙15 |
| Inotrope score | 8∙98 +/-0∙85 | 31∙72 +/-4∙74 |
| PELOD Day 1 | 10∙43 +/-0∙24 | 16 +/-0∙94 |
| Lactate | 1∙61 +/-0∙05 | 2∙99 +/-0∙45 |
| Base excess | -1∙92 +/-0∙21 | -4∙31 +/-0∙67 |
| Platelet | 215∙56 +/-5∙52; | 164∙16 +/-20∙99 |
| pNGAL | 242∙95 +/-17∙82 | 432∙79 +/-89∙76 |
| Procalcitonin | 8∙49 +/-1∙82 | 70∙97 +/-33∙76 |

A logistic regression model was fitted to RRT as an outcome and PELOD, bypass time, lactate, procalcitonin, and age were included in the best fit model. The coefficients, odds ratio, and 95% confidence interval were estimated (S4 Table). For the continuous variable age; the coefficient is negative, indicating that the younger the patient, the lower the probability of RRT which is the opposite pattern of the other variables in the model. The odds ratio provides the odds of a patient having RRT for each unit increase in that individual variable. Using this model the AUC was 0∙88, suggesting a good fit (S1 Fig).

**S1 Fig. ROC curve of the model of best fit for RRT**

**
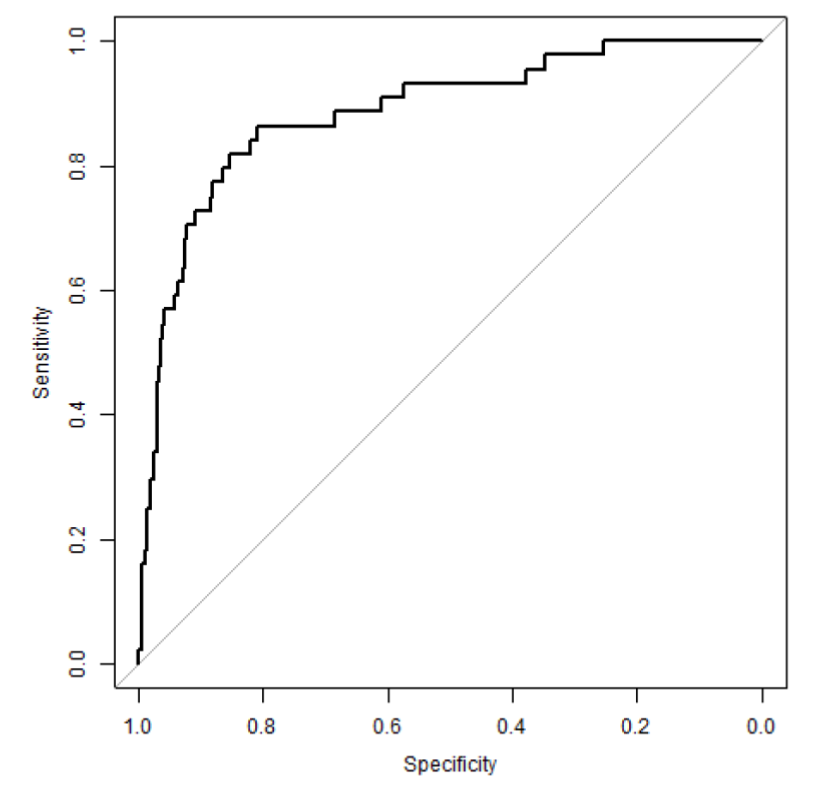
**

**S4 Table. Final model of best fit for RRT**

|  | Coefficient | Standard Error of the mean | Odds Ratio | OR 95% CI | P value |
| --- | --- | --- | --- | --- | --- |
| Intercept | -5∙83 | 0∙66 | 0∙003 | (0∙0007,  0∙01) |  |
| PELOD | 0∙15 | 0∙03 | 1∙17 | (1∙10,  1∙24) | <0∙001 |
| Bypass time | 0∙01 | 0∙003 | 1∙01 | (1∙00,  1∙02) | 0∙001 |
| Lactate | 0∙23 | 0∙08 | 1∙26 | (1∙07,  1∙48) | 0∙004 |
| Procalcitonin | 0∙01 | 0∙001 | 1∙01 | (1∙00,  1∙01) | 0∙0009 |
| Age | -0∙14 | 0∙06 | 0∙87 | (0∙75,  0∙97) | 0∙0265 |

**Development of a biomarker model for RRT**

Initial and maximal blood biomarker data were included in a best fit model of RRT incidence. A cox-proportional hazards regression algorithm, with time-varying covariates, was used to compare longitudinal biomarker data. The initial biomarkers model used uNGAL and surgery (none, cardiac, or other) (S5 Table), whilst the maximal values model, included uNGAL, urea, creatinine, and surgery (S6 Table). A log transformation to base 2 was used to aid interpretation of the coefficients and improve fit. The exponentiated coefficient is the odds ratio of doubling the predictor variable, for instance, doubling uNGAL increases the odds of receiving RRT by 1∙26 times. Both maximal urea and creatinine concentrations have a positive coefficient, indicating an increased risk as concentrations increase. Additionally, the categorical variable of cardiac surgery increased the patient’s risk of RRT but this was expressed as differential reductions in risk in those without surgery as compared to those undergoing non-cardiac surgery. For each model, ROC curves were used to identify the optimal threshold (S2 Fig). As shown in S7 Table, AUC is higher for maximal values; 0∙89 compared to 0∙72 for initial biomarker values.

**S2 Fig. ROC curve of RRT modelling.** Using A: first recorded values B: maximal blood biomarker values


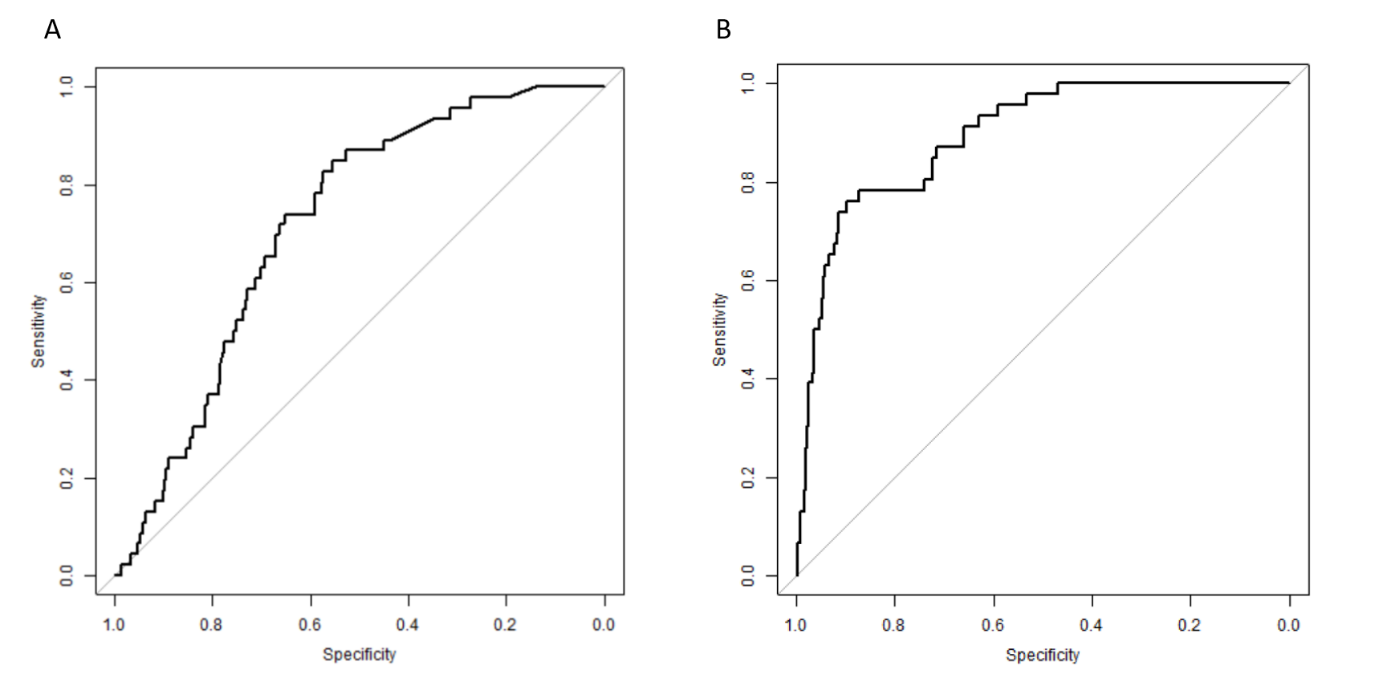


**S5 Table. Model of RRT using initial recorded values of the blood biomarker data**

| First Recorded values | Coefficient | Standard Error of the mean | Odds Ratio | OR 95% CI | P value |
| --- | --- | --- | --- | --- | --- |
| Intercept | -3∙66 | 0∙47 | 0∙03 | 0∙01, 0∙06 | - |
| Log2(uNGAL) | 0∙23 | 0∙06 | 1∙26 | 1∙11, 1∙43 | 0∙0002 |
| No Surgery | -1∙25 | 0∙49 | 0∙29 | 0∙10, 0∙69 | 0∙0112 |
| ‘Other’ Surgery | -0∙71 | 0∙50 | 0∙49 | 0∙16, 1∙21 | 0∙159 |

**S6 Table. Model of RRT using maximal recorded values of the blood biomarker data**

| Maximum values | Coefficient | Standard Error of the mean | Odds Ratio | OR 95% CI | P value |
| --- | --- | --- | --- | --- | --- |
| Intercept | -12∙39 | 1∙61 | 0 | 0 | - |
| Log2(MaxUrea) | 1∙11 | 0∙33 | 3∙04 | 1∙61, 5∙98 | 0∙0009 |
| Log2(MaxCreat) | 0∙86 | 0∙34 | 2∙35 | 1∙22, 4∙60 | 0∙011 |
| Log2(MaxUNGAL) | 0∙22 | 0∙08 | 1∙24 | 1∙06, 1∙47 | 0∙010 |
| No Surgery | -1∙84 | 0∙63 | 0∙16 | 0∙04, 0∙49 | 0∙003 |
| ‘Other’ Surgery | -1∙27 | 0∙66 | 0∙28 | 0∙07, 0∙93 | 0∙053 |

**S7 Table. ROC analysis for the RRT using blood biomarker data**

| **Model**  **(%)** | **AUC** | **95% CI** | **Optimal Threshold** | **Sensitivity** | **Specificity** | **Positive predictive value** | **Negative predictive value** |
| --- | --- | --- | --- | --- | --- | --- | --- |
| First recorded values | 0.72 | 0.66-0.80 | 7∙7 | 0.74 | 0.65 | 0.15 | 0.97 |
| Maximum values | 0.89 | 0.85-0.94 | 11∙6 | 0.78 | 0.87 | 0.34 | 0.98 |
