# Supplementary material for: Identifying critically ill children at high risk of acute kidney injury and renal replacement therapy
Source: PLoS One. 2020 Oct 29;15(10):e0240360. doi: 10.1371/journal.pone.0240360 (PMC7595286; doi:10.1371/journal.pone.0240360)
Supplement: Attachment — Submitted filename: McGalliard PLOS One response to reviewers comments Final.docx [file pone.0240360.s004.docx]

PONE-D-20-09458
Identifying critically ill children at high risk of acute kidney injury and renal replacement therapy
PLOS ONE

Dear Dr. McWilliam,

Thank you for submitting your manuscript to PLOS ONE. After careful consideration, we feel that it has merit but does not fully meet PLOS ONE’s publication criteria as it currently stands. Therefore, we invite you to submit a revised version of the manuscript that addresses the points raised during the review process.

Please submit your revised manuscript by 6 weeks of receipt of this message. If you will need more time than this to complete your revisions, please reply to this message or contact the journal office at [plosone@plos.org](mailto:plosone@plos.org). When you're ready to submit your revision, log on to <https://www.editorialmanager.com/pone/> and select the 'Submissions Needing Revision' folder to locate your manuscript file.

Please include the following items when submitting your revised manuscript:

- A rebuttal letter that responds to each point raised by the academic editor and reviewer(s). You should upload this letter as a separate file labeled 'Response to Reviewers'.
- A marked-up copy of your manuscript that highlights changes made to the original version. You should upload this as a separate file labeled 'Revised Manuscript with Track Changes'.
- An unmarked version of your revised paper without tracked changes. You should upload this as a separate file labeled 'Manuscript'.

If you would like to make changes to your financial disclosure, please include your updated statement in your cover letter. Guidelines for resubmitting your figure files are available below the reviewer comments at the end of this letter.

If applicable, we recommend that you deposit your laboratory protocols in protocols.io to enhance the reproducibility of your results. Protocols.io assigns your protocol its own identifier (DOI) so that it can be cited independently in the future. For instructions see: <http://journals.plos.org/plosone/s/submission-guidelines#loc-laboratory-protocols>

We look forward to receiving your revised manuscript.

Kind regards,

Rajendra Bhimma, PhD
Academic Editor
PLOS ONE

Thank you for this review. Please find our responses in red text below.

Journal Requirements:

When submitting your revision, we need you to address these additional requirements.

1. Please ensure that your manuscript meets PLOS ONE's style requirements, including those for file naming. The PLOS ONE style templates can be found at <https://journals.plos.org/plosone/s/file?id=wjVg/PLOSOne_formatting_sample_main_body.pdf> and <https://journals.plos.org/plosone/s/file?id=ba62/PLOSOne_formatting_sample_title_authors_affiliations.pdf>

The manuscript has been updated

2. Thank you for including your ethics statement:

"Ethics approval was granted by the local UK research ethics committee (REC
10/H1014/52). Written, informed consent was gained from the primary care giver.

Please amend your current ethics statement to include the full name of the ethics committee/institutional review board(s) that approved your specific study.

Once you have amended this statement in the Methods section of the manuscript, please add the same text to the “Ethics Statement” field of the submission form (via “Edit Submission”).

For additional information about PLOS ONE ethical requirements for human subjects research, please refer to <http://journals.plos.org/plosone/s/submission-guidelines#loc-human-subjects-research>.

This has been updated

3. We note that you have indicated that data from this study are available upon request. PLOS only allows data to be available upon request if there are legal or ethical restrictions on sharing data publicly. For information on unacceptable data access restrictions, please see <http://journals.plos.org/plosone/s/data-availability#loc-unacceptable-data-access-restrictions>.

In your revised cover letter, please address the following prompts:

a) If there are ethical or legal restrictions on sharing a de-identified data set, please explain them in detail (e.g., data contain potentially identifying or sensitive patient information) and who has imposed them (e.g., an ethics committee). Please also provide contact information for a data access committee, ethics committee, or other institutional body to which data requests may be sent.

b) If there are no restrictions, please upload the minimal anonymized data set necessary to replicate your study findings as either Supporting Information files or to a stable, public repository and provide us with the relevant URLs, DOIs, or accession numbers. Please see <http://www.bmj.com/content/340/bmj.c181.long> for guidelines on how to de-identify and prepare clinical data for publication. For a list of acceptable repositories, please see <http://journals.plos.org/plosone/s/data-availability#loc-recommended-repositories>.

We will update your Data Availability statement on your behalf to reflect the information you provide.

We have updated our data availability statement as follows:

‘Data can be made available upon reasonable request to the senior author of this paper (EDC, edcarrol@liverpool.ac.uk). This request will need to include a formal request for collaboration, a study protocol, and clearly outlined study objectives. The decision to share data will lie with the University of Liverpool. A formal data sharing agreement will need to be agreed and signed prior to any (de-identified) data being exchanged.’

Additional Editor Comments (if provided):

Thank you very much for submitting your manuscript to PLOS ONE. Following a detailed review of the manuscript by three reviewers, there have been several comments raised by the reviewers that need to be addressed (see below). Please do this in accordance with the requirements of the journal.


[Note: HTML markup is below. Please do not edit.]

Reviewers' comments:

Reviewer's Responses to Questions

**Comments to the Author**

1. Is the manuscript technically sound, and do the data support the conclusions?

The manuscript must describe a technically sound piece of scientific research with data that supports the conclusions. Experiments must have been conducted rigorously, with appropriate controls, replication, and sample sizes. The conclusions must be drawn appropriately based on the data presented.

Reviewer #1: Yes

Reviewer #2: Yes

Reviewer #3: No

2. Has the statistical analysis been performed appropriately and rigorously?

Reviewer #1: Yes

Reviewer #2: Yes

Reviewer #3: No

3. Have the authors made all data underlying the findings in their manuscript fully available?

The [PLOS Data policy](http://www.plosone.org/static/policies.action#sharing) requires authors to make all data underlying the findings described in their manuscript fully available without restriction, with rare exception (please refer to the Data Availability Statement in the manuscript PDF file). The data should be provided as part of the manuscript or its supporting information, or deposited to a public repository. For example, in addition to summary statistics, the data points behind means, medians and variance measures should be available. If there are restrictions on publicly sharing data—e.g. participant privacy or use of data from a third party—those must be specified.

Reviewer #1: Yes

Reviewer #2: Yes

Reviewer #3: No

4. Is the manuscript presented in an intelligible fashion and written in standard English?

PLOS ONE does not copyedit accepted manuscripts, so the language in submitted articles must be clear, correct, and unambiguous. Any typographical or grammatical errors should be corrected at revision, so please note any specific errors here.

Reviewer #1: No

Reviewer #2: Yes

Reviewer #3: No

5. Review Comments to the Author

Please use the space provided to explain your answers to the questions above. You may also include additional comments for the author, including concerns about dual publication, research ethics, or publication ethics. (Please upload your review as an attachment if it exceeds 20,000 characters)

Reviewer #1: McGalliard and colleagues perform a prospective analysis of the ability of uNGAL and pNGAl to predict SCr based KDIGO Stage 2 AKI in critically ill children. They then assessed the ability of the RAI to do the same, and then combined the RAI and NGAL levels to determine if the performance improved. Then then undertook an extensive assessment of multiple factors to predict RRT provision in the cohort. The authors found that combination of the RAI and Day uNGAL provided the best predictive performance. The following issues require attention:


1) The section developing the model for RRT prediction can be shortened signficantly. I find it to be distracting from the overall message, and in fact, is not even mentioned in the abstract. As the authors note, RRT provision is a soft outcome measure, as indications for RRT provision were not provided in the protocol. I would recommend significant shorterning of this part of the manuscript, or removing it altogether.

The section of the Results describing the development of the RRT prediction model and associated tables and figures has been largely removed and will be provided as supplementary material. A paragraph describing the incidence and outcomes of RRT is retained in the main manuscript.

2) There are two distinct cohorts in this population -- a group of heterogenous PICU patients and post-cardiac surgery patients. The RAI has never been validated in the post-cardiac surgery patients. It would be helpful for the authors to do a sensitivity analysis of RAI and NGAL performance in these two cohorts separately.

Thank you for your comment. We have completed an additional analysis in the sub-population of 360 post-cardiac surgery patients. The results section has been updated to include these results.

3) The authors define AKI as Stage 2 AKI--this should be called severe AKI or Stage 2 AKI throughout.

This has been amended throughout.

4) The authors need to state when the RAI was calculated. In the studies cited in there references, it was calcuated at 12 hours of PICU admission.

The RAI was calculated retrospectively using data obtained upon admission to PICU, and their first serum creatinine measurement on their first day in PICU. The text has been updated to include this detail.

5) I am confused by the UOP criteria---were they used or not? In the Methods, the authros state they were (although the criteria they cite is Stage 1 AKI by KDIGO, this needs to be reconciled), but in the Discussion they state they don't have data on UOP? If they did use UOP, they need to use the correct KDIGO staging for Stage 2, and in the results, theshould note how manay patients had Stage 2 AKI by SCr, UOP or both.

Apologies for the confusion. Urine output data was not collected, and was therefore not used for defining AKI. The text has been amended to clarify this.

6) A substantial percentage of patients were excluded because they did not have a reference SCr in the 3 months prior to PICU admission. Numerous pediatric studies, including AWARE have imputed baseline SCr in such cases based on the method of Zappitelli. The authors could also use the Pottel method. In any case, these patients should be assessed.

We have added a paragraph to the Results section to address this issue. The Pottel method has been applied as height data were not collected.

Minor

1) Data is the plural form of datum.

This has been amended throughout.

2) The authors do not need to state a difference is "significant", that is implied by the methods. If the AKI group has a higher value, then just state, "Patients with AKI has a longer duration of PICU stay", for example.

This has been amended throughout.

3) Please note the first mortality variable in the AKI comparision table is PICU mortality.

This has been amended.

Reviewer #2: A question to the authors: What are the costs for the tests especially if they were point of care tests which could determine if the child was in established AKI and if so to commence renal replacement therapy sooner rater than later.i.e. would it be cost effective done for the right indication?

Thank you for your question. We are not in a position to perform a health economic analysis of this approach at present. Costs for the ARCHITECT NGAL test used are available in a recent NICE publication at <https://www.nice.org.uk/guidance/dg39/chapter/3-Evidence>

Reviewer #3: A prospective observational study was conducted to assess the ability of biomarkers, neutrophil gelatin-aseassociated lipocalin in urine (uNGAL) and plasma (pNGAL), and renal angina index (RAI) to predict acute kidney injury (AKI). The AUC of ROC curves were used to identify the best cut-points in biomarkers. The manuscript requires further clarification of the methods and results before drawing conclusions.

Major revision:
1- The manuscript lacks clarity and organization. The methods and results have not been clearly explained.

Thank you for your comments. We have taken the opportunity to significantly review the manuscript, taking into account this and your other comments. We hope you will find the clarity and organisation of the manuscript improved.

2- Clearly indicate how RAI and uNGAL/pNGAL values were used to calculate the sensitivity, specificity, PPV and NPV shown in table 4.

Sensitivity and specificity was calculated for a range of cut-off values for uNGAL and pNGAL. Sensitivity and specificity curves were then plotted on the same graph and the point of crossover is the cut-off value that optimises sensitivity and specificity. This cut-off value was then used to classify patients as being positive above the cut-off or negative equal to or below the cut-off. The cut-offs, sensitivity etc. are reported in table 4.

3- Further clarify the statistical analysis section by providing details of the receiver operating characteristic curve.

The ROC curve is calculated by plotting sensitivity against 1-specificity for a range of cut-off values. The closer the area under the ROC is to one the better the screening tool is at discriminating between positive and negative cases.

Minor revisions:
1- Abstract: More fully develop the abstract to help clarify the purpose, statistical methods and results of the study.

The abstract has been reviewed in order to address these aspects.

2- Line 135: Chi-square tests were used to COMPARE categorical variables.

This has been amended.

3- Line 138: Clarify the sentence, “Logistic regression and Cox proportional-hazards models were fitted to the outcome renal replacement therapy, using the independent variables where an association was found in the univariate analysis.” Were MULTIVARIATE logistic and Cox proportional-hazard models fitted?

Multivariate logistic regression and Cox proportional-hazard models were fitted.

4- Check the line spacing of results in Table 1, specifically for Renal Replacement Therapy.

This has been amended.

5- Table 5: Clarify if standard error refers to the standard error of the mean.

The standard errors do refer to the standard errors of the mean. The relevant sections (now moved to supplementary) have been updated to clarify this.

6. PLOS authors have the option to publish the peer review history of their article ([what does this mean?](https://journals.plos.org/plosone/s/editorial-and-peer-review-process#loc-peer-review-history)). If published, this will include your full peer review and any attached files.


If you choose “no”, your identity will remain anonymous but your review may still be made public.


**Do you want your identity to be public for this peer review?** For information about this choice, including consent withdrawal, please see our [Privacy Policy](https://www.plos.org/privacy-policy).

Reviewer #1: Yes: Stuart L Goldstein

Reviewer #2: No

Reviewer #3: No


[NOTE: If reviewer comments were submitted as an attachment file, they will be attached to this email and accessible via the submission site. Please log into your account, locate the manuscript record, and check for the action link "View Attachments". If this link does not appear, there are no attachment files.]

While revising your submission, please upload your figure files to the Preflight Analysis and Conversion Engine (PACE) digital diagnostic tool, <https://pacev2.apexcovantage.com/>. PACE helps ensure that figures meet PLOS requirements. To use PACE, you must first register as a user. Registration is free. Then, login and navigate to the UPLOAD tab, where you will find detailed instructions on how to use the tool. If you encounter any issues or have any questions when using PACE, please email PLOS at [figures@plos.org](mailto:figures@plos.org). Please note that Supporting Information files do not need this step.
